# Internet Use for Obtaining Medicine Information: Cross-sectional Survey

**DOI:** 10.2196/40466

**Published:** 2023-02-02

**Authors:** Trine Strand Bergmo, Vilde Sandsdalen, Unn Sollid Manskow, Lars Småbrekke, Marit Waaseth

**Affiliations:** 1 Norwegian Centre for E-health Research University Hospital of North Norway Tromsø Norway; 2 Department of Pharmacy UiT The Artic University of Norway Tromsø Norway

**Keywords:** credibility, credible, cross-sectional, eHealth, health information, information behavior, information retrieval, information science, information seeking, internet, medication, medicine information, misinformation, patient education, pharmaceutical, pharmacist, pharmacy, survey, trust, web-based information

## Abstract

**Background:**

The internet is increasingly being used as a source of medicine-related information. People want information to facilitate decision-making and self-management, and they tend to prefer the internet for ease of access. However, it is widely acknowledged that the quality of web-based information varies. Poor interpretation of medicine information can lead to anxiety and poor adherence to drug therapy. It is therefore important to understand how people search, select, and trust medicine information.

**Objective:**

The objectives of this study were to establish the extent of internet use for seeking medicine information among Norwegian pharmacy customers, analyze factors associated with internet use, and investigate the level of trust in different sources and websites.

**Methods:**

This is a cross-sectional study with a convenience sample of pharmacy customers recruited from all but one community pharmacy in Tromsø, a medium size municipality in Norway (77,000 inhabitants). Persons (aged ≥16 years) able to complete a questionnaire in Norwegian were asked to participate in the study. The recruitment took place in September and October 2020. Due to COVID-19 restrictions, social media was also used to recruit medicine users.

**Results:**

A total of 303 respondents reported which sources they used to obtain information about their medicines (both prescription and over the counter) and to what extent they trusted these sources. A total of 125 (41.3%) respondents used the internet for medicine information, and the only factor associated with internet use was age. The odds of using the internet declined by 5% per year of age (odds ratio 0.95, 95% CI 0.94-0.97; *P=*.048). We found no association between internet use and gender, level of education, or regular medicine use. The main purpose reported for using the internet was to obtain information about side effects. Other main sources of medicine information were physicians (n=191, 63%), pharmacy personnel (n=142, 47%), and medication package leaflets (n=124, 42%), while 36 (12%) respondents did not obtain medicine information from any sources. Note that 272 (91%) respondents trusted health professionals as a source of medicine information, whereas 58 (46%) respondents who used the internet trusted the information they found on the internet. The most reliable websites were the national health portals and other official health information sites.

**Conclusions:**

Norwegian pharmacy customers use the internet as a source of medicine information, but most still obtain medicine information from health professionals and packet leaflets. People are aware of the potential for misinformation on websites, and they mainly trust high-quality sites run by health authorities.

## Introduction

The internet is a central source of health-related information, and more than three-quarters of the population in Europe use the internet for health purposes [1,2]. A recent study from 2020 found that nearly 6 in 10 older women with chronic illnesses used the internet to obtain health information [3]. People want information to facilitate decision-making and self-management [4], and they tend to prefer the internet for ease of access [5,6].

Over the past two decades, the patient-doctor relationship has shifted from paternalistic to more shared decision-making and responsibilities [7,8]. The increased participation and responsibilities for one’s own health and medical treatment [7,9,10] has created a drive for consumer-focused and patient-centered health care models [11-13]. Person-centered health care models promote self-management, which refers to any action taken to recognize, treat, and manage one’s own health or chronic conditions [10]. Making informed decisions requires access to high-quality information [14-16].

Patients have a key role in safe and effective medicine use [17,18]. They need basic information about how the medicine works, how to use it, potential benefits and harms, and how it is compared with other available treatment options or the option not to treat [15,19]. Well-designed and well-written medicine information can improve knowledge and contribute to informed choices about medicine use and thereby improve health [20]. In the past, the only source of written medicine information was the label on the medicine package [20]. Since then, there has been a gradual increase in regulated information provided to patients. The information became available first on paper as advertisements, then in package leaflets, and later via the internet, smartphone apps, and other electronic devices [21]. The driving forces for better access to medicine information have been drug safety, people’s right to information on their own treatment, and motivating people to take more responsibility for their own health [22].

Several studies have found that patients mainly get medicine information from physicians, pharmacists, and package leaflets [17,21,23-25]. Most people prefer to get information from a health professional in person [5,26-29], and they tend to trust physicians, pharmacists, and package leaflets the most [5,29-31]. However, the leaflet can also be discouraging as some find the information difficult to understand and therefore rely on other alternatives [32].

The use of the internet as a source of medicine information has increased over the past decades. A repeated population study from Finland found that the proportion of people using the internet to obtain medicine information increased from 1% in 1999 to 16% in 2014 [21]. Studies from other countries have reported internet use for medicine information ranging from 3% to 29% in the 2000s [31,33-36]. A more recent study from 2019 reported that 37% used the internet for medicine information [6], while 68% of the internet users searched for medicine information on the internet [24]. Internet users are usually younger well-educated women, and they tend to use national health portals and pharmacy websites to look for medicine information [24,27].

As anyone can easily develop a website, internet-based health information can be of varying quality [36,37]. Several studies have found nonvalidated, inaccurate, and biased web-based health information sources [37-40]. Studies have also found that using the internet for medicine information can create more concerns and uncertainty about medicine use [41,42]. Inconsistency between different web-based sources has also been reported [11,43]. Since medicine information can influence views and medicine use behavior [44,45], support tools and skills in how to search and interpret web-based medicine information is crucial [45-48]. Even if people express concerns about the quality of internet-based medicine information, the convenience of access to this information seems to outweigh these reservations [49]. Since people frequently access health information on the internet, ensuring available, valid, usable, and high-quality information should be a priority [5].

To our knowledge, there are no studies investigating the extent of internet use to obtain medicine information in Norway. There are also few recent international studies on how much the medicine users trust the medicine information found on the internet and which sites they trust the most.

The main objective of this study was to establish the extent of internet use for seeking medicine information. Secondary objectives were to identify factors associated with internet use, describe the use of other sources for medicine information, and investigate the level of trust in different information sources and websites.

## Methods

### Study Design and Setting

This is a cross-sectional study with a convenience sample of pharmacy customers recruited from community pharmacies in Tromsø, a medium size municipality in Norway (77,000 inhabitants). Persons (aged ≥16 years) able to complete a questionnaire in Norwegian were asked to participate in the study. The study aimed to include respondents during September and October 2020. Due to COVID-19 restrictions, social media was also used to recruit medicine users.

### Questionnaire Development

The research team developed a structured questionnaire to collect views on the internet as a source of medicine information. The questionnaire included 12 questions, and the first 7 collected background variables such as age, gender, education, and rural or urban area of residence. We also asked if the respondents were health professionals or not (yes/no). Questions about the number of medicines used during the previous week (none, 1-4, 5-7, 8-10, and ≥10) and if they used regular prescription medicines (yes/no) were added. Regular medicine use was defined as answering yes to the following question: “Do you use prescription medicines regularly? This includes tablets, mixtures, medical creams, eye drops, etc.”

The main questions have been validated in previous research from Finland [21,31]. “In the past 12 months, from which sources did you receive information on the medicine you have been using (both prescription and self-medication)?” The question was followed by a list of available options: physician, pharmacist, package leaflet, nurses, relatives/friends, advertising, and the internet. The respondents could choose more than one option. Those who had chosen the internet option were further asked “In the last 12 months, which websites have you used to obtain medicine information?” Available options provided were the Norwegian public health portal (Helsenorge.no), other official health information sites, pharmacy websites, web-based questions and answers from health professionals, general health and lifestyle sites, and social media. The respondents could include other sources than those provided in the lists above as free text. An additional question registered how much they trusted the web-based sources they used, both in general and for the specific websites listed: “How much do you trust the medicine information you find on the internet/specified website?” Participants were also asked to indicate their level of trust in health personnel as a source of medicine information for comparison: “How much do you trust the medicine information you receive from health personnel?” Lastly, the respondents were asked to indicate why they were searching for medicine information with the following options: to acquire information on how the medicine works, possible side effects, how to take the medicine, or the recommended dosing, or they received insufficient information from health personnel.

The questionnaire was piloted to assess the face and content validity. A total of 8 persons assessed the questionnaire for clarity, ease of completion, and functionality. No modifications were required after the piloting. The pilot data were not included in the analysis.

### Main Outcome Measures

The main outcome measures were the proportion of respondents who reported using the different medicine information sources, the level of trust in the different sources, and the proportion of respondents reporting reasons for seeking information. The participants rated the level of trust in the different sources and websites using a 4-point scale: completely trustworthy, somewhat trustworthy, not much trustworthy, and not trustworthy.

### Survey Distribution

Managers at 12 pharmacies were asked if they would allow distribution of questionnaires at their premises. Eleven pharmacies agreed to participate, and the questionnaires were distributed to customers in person by one of the researchers (VS). The researcher invited customers to complete a 4-page questionnaire. Those who consented to participate received an information sheet and the questionnaire. The questionnaire was available both on paper and as a web-based version. They were asked to return the completed questionnaire in a prepaid envelope or to complete the survey on the internet. The link to the web-based form was included in the information sheet. They also had the opportunity to complete the questionnaire at the pharmacy. The questionnaires were numbered to keep track of the number of respondents and avoid duplicates. Customers who declined to participate were registered as such. We report the response rate as defined by American Association for Public Opinion Research [50].

After 6 weeks of data collection, the distribution of questionnaires in the pharmacies was stopped due to new COVID-19 restrictions, but we continued distributing questionnaires using social media. Only those who confirmed that they had used a prescription or nonprescription medicine during the last week were included via social media (n=15).

### Data Analyses

Data were analyzed using IBM SPSS Statistic 25 (IBM Corp). Demographics and responses to the questions about the medicine information sources used, reasons for use, and trust were analyzed using descriptive statistics. Pearson ^2^ test was used to explore univariate association between categorical variables, whereas *t* test or Mann-Whitney test was used for continuous variables depending on their distribution. We used logistic regression to assess the association of explanatory variables and the use of the internet for medicine information. We included age, gender, education, urban and rural residence, whether the respondents were health personnel, and if they used regular prescription medicines as independent variables. We used a stepwise model with backward selection to decide the final model. The results are presented as odds ratio (OR) and 95% CI. A *P* value of ≤.05 was considered statistically significant.

### Ethics Approval

The Data Protection Officer at the University Hospital of North Norway approved the study protocol (Project No. 02578). The participants received both written and oral information about the study. Completing the questionnaire was considered as giving informed consent. We included persons aged ≥16 years as they can buy medicines, fill prescriptions, and give informed consent to participate in surveys. The data were handled according to local security requirements. We used the “Good practice in the conduct and reporting of survey research”—a checklist by Kelley et al [51]—to ensure structure and style of this manuscript.

## Results

A total of 414 persons were invited to participate in the survey, and 303 (73.2%) accepted and completed the questionnaire, 228 used the paper questionnaire, and 75 used the web-based form.

The mean age of the respondents was 51 (range 16-92) years. A total of 125 (41.3%) respondents were older than 60 years, and 76 (25%) were older than 70 years (Table 1). Two-thirds (n=196, 64.6%) of the respondents were women and 62.7% (n=190) held a college or university degree. Note that 245 (80.8%) respondents had used at least one medicine during the last week (prescription and over-the-counter medicine), and 210 (69.3%) used regular prescription medicines. Almost one-third (n=88, 29%) of the respondents were health personnel.

A total of 125 (41.3%) respondents used the internet as a source of medicine information, and the internet users were younger, had a college or university degree, and were health professionals (see Tables 1 and 2). The main medicine information topics sought through the internet were side effects (n=106, 84.8% of the internet users), how the medicine works (n=82, 66%), and how to take the medicine (n=56, 45%; data not shown in Table 1). Only 6 (4.8%) obtained information about interactions.

A logistic regression model shows that age was the only factor associated with using the internet as a medicine information source (see Table 2). It was an inverse association between age and the odds of using the internet (OR 0.95, 95% CI 0.94-0.97; *P*=.048). The odds of internet use were 5% lower per 1-year of higher age. Respondents using regular prescription medicines also tended to use the internet more, but this association was not statistically significant. Gender, urban or rural residence, and whether the respondents were health personnel did not make a significant contribution and were excluded in the final regression model.

**Table 1:** Respondents’ characteristics presented by internet and no internet use for seeking medicines information.

Respondents’ characteristics			Total (N=303)		Internet use (n=125)		No internet use (n=178)		*P* value	
**Gender, n (%)**										.83
	Female	196 (64.6)		85 (68)		116 (65.2)				
	Male	107 (35.3)		40 (32)		62 (35)				
Age (years), mean (SD)			51.5 (21.2)		40.9 (18.9)		58.9 (19.7)		.45	
**Age groups (years), n (%)**										N/A^a^
	16-30	94 (31)		64 (39)		30 (55)				
	31-40	17 (6)		6 (7)		11 (10)				
	41-50	30 (24)		18 (12)		12 (18)				
	51-60	37 (12)		11 (15)		26 (22)				
	61-70	49 (16)		14 (20)		35 (29)				
	>70	76 (25)		12 (32)		64 (44)				
**Education** **, n (%)**										.03
	High school or less	113 (37.3)		35 (28)		78 (44)				
	College or university	190 (62.7)		90 (72)		100 (56.2)				
**Residency, n (%)**										.06
	Urban	257 (84.8)		105 (84.0)		152 (85.4)				
	Rural	46 (15)		20 (16)		26 (15)				
**Health professional, n (%)**										.02
	Yes	88 (29)		48 (38)		40 (22)				
	No	213 (70.3)		76 (62)		138 (77.5)				
**Number of drugs used last week, n (%)**										.09
	0	57 (19)		18 (14)		39 (22)				
	1-4	190 (62.7)		89 (71)		101 (56.7)				
	5-7	46 (15)		15 (12)		31 (17)				
	8-10	7 (2)		3 (2.2)		4 (2.5)				
	>10	2 (0.7)		0 (0)		2 (1.5)				
	Do not want to answer	1 (0.3)		1 (0.8)		0 (0)				
**Regular prescription drugs, n (%)**										.57
	Yes	210 (69.3)		86 (69)		124 (69.6)				
	No	90 (30)		37 (30)		53 (29.4)				
	Do not want to answer	3 (1)		2 (1)		1 (0.6)				

^a^N/A: not applicable.

**Table 2:** Internet use for medicine information and associated factor.

		Unadjusted effects		Adjusted effects^a^	
		OR^b^ (95% CI)	*P* value	OR (95% CI)	*P* value
Age (years)		0.96 (0.94-0.97)	.03	0.95 (0.94-0.97)	.048
**Education**			.04		.13
	College or university^c^	1.00		1.00	
	High school or less	0.49 (0.30-0.81)		0.65 (0.38- 1.13)	
**Regular prescription medicine use**			.98		.06
	Yes	0.99 (0.60-1.94)		1.76 (0.99-3.15)	
	No^c^	1.00		1.00	

^a^Pseudo *R*^2^=0.150.

^b^OR: odds ratio.

^c^Reference in the model.

Almost half (n=58, 46%) of the internet users (n=125) trusted the medicine information they obtained from the internet. A higher proportion of the women trusted the internet compared to the men (*χ*^2^_4_=10.3; *P*=.03).

The websites regarded as most trustworthy were the national health portals and other official health information sites (see Table 3). These were also the most frequently used sources. Public health portal sites were considered completely trustworthy by 75 (60%) respondents, while 22 (18%) respondents considered them somewhat trustworthy. Other health- and medicine-related sites run by governmental organizations were also considered completely trustworthy by 60 (48%) and somewhat trustworthy by 28 (22%) respondents. Only 3 (2%) respondents found social media completely trustworthy.

Noninternet sources used to obtain medicine information are shown in Table 4. The main sources were physicians (n=191, 63%), pharmacists (n=142, 46.9%), and package leaflets (n=124, 40.9%). A total of 36 (12%) had not obtained medicine information during the last 12 months.

**Table 3:** Level of trust in internet sites used for obtaining medicine information (% of total internet users).

Internet sites		Level of trust (n=125)			
		Completely trustworthy	Somewhat trustworthy	Not much trustworthy	Not trustworthy
**Helsenorge.no^a^,** **n (%)**		75 (60)	22 (18)	3 (2)	3 (2)
	My electronic prescriptions	65 (52)	16 (13)	7 (6)	7 (6)
	Summary care record	43 (34)	13 (10)	6 (4)	6 (5)
	My hospital health record	38 (30)	19 (15)	9 (7)	9 (7)
Physician’s desktop reference, n (%)		60 (48)	28 (22	4 (3)	7 (5)
Pharmacy websites, n (%)		32 (26)	47 (38)	2 (1.6)	4 (3.2)
Web-based questions/answered by health professionals, n (%)		19 (15)	55 (44)	34 (27)	38 (30)
General health and lifestyle, n (%)		1 (0.8)	7 (6)	17 (14)	44 (35)
Social media, n (%)		3 (2)	5 (4)	15 (12)	66 (52)

^a^Helsenorge.no is a national health portal established in 2011 providing comprehensive health information and eHealth services to the citizens. The portal includes information about both current and past prescriptions in my prescriptions, the summary care record, and my hospital record.

**Table 4:** Use of medicine information sources across gender, age group (≤65 years), regular medicine use, and health professionals (can use more than one source).

Sources	Total (N=303), n (%)	Women (n=196), n (%)	Aged 16-65 years (n=196), n (%)	Regular medicine use (n=210), n (%)	Health professionals (n=88), n (%)
Physicians	191 (63.0)	126 (64.3)	117 (59.7)	151 (71.9)^a^	45 (51)^a^
Pharmacists	142 (46.9)	108 (55.1)^a^	108 (55.1)^a^	111 (52.8)^b^	56 (64)^a^
Package leaflets	128 (40.9)	94 (48)^a^	92 (47)^b^	99 (47)^b^	44 (50)
Internet	125 (41.3)	80 (41)	105 (54)^a^	86 (41)	48 (54)^a^
Relatives/friends	31 (10)	18 (9)	25 (13)	20 (9)	8 (9)
Advertisements	8 (3)	6 (3)	6 (3)	5 (2)	2 (2)
Other^c^	19 (7)	13 (7)	16 (8)	13 (6)	13 (15)^c^
No use of MI^d^ sources last year	36 (12)	20 (10)	19 (10)	11 (5)	11 (12)

^a^*P*<.01.

^b^*P*<.05.

^c^Other includes nurses, being health personnel themselves, and physician’s desktop reference.

^d^MI: medicine information.

Univariate *χ*^2^ analyses showed that a higher proportion of women obtained medicine information from pharmacists and package leaflets than men, and a higher proportion of women younger than 65 years used pharmacists, packet leaflets, and the internet compared to women older than 65 years. Furthermore, a higher proportion of persons with regular prescriptions used physicians, pharmacists, and packet leaflets compared to those without regular prescriptions. Health professionals used physicians, pharmacists, and the internet more than persons without a health professional background.

A total of 275 (90.8%) respondents trusted health professionals as a source and women trusted them more than men (*χ*^2^_4_=7.7; *P*=.03; data not shown). The respondents who had sought medicine information mainly used 1-3 sources to obtain information (n=224, 73.9% of the respondents), while 43 (14%) used more than 3 sources.

## Discussion

### Principal Findings

This study found that 125 (41.3%) respondents used the internet to obtain medicine information, and the only factor associated with internet use was age. The odds of using the internet as a source was inversely associated with age and declines 5% per year increase in age. The model showed no association between internet use and gender, level of education, regular medicine use, residency, or if they were health professionals. The main purpose for using the internet was to obtain information about side effects. Other sources of medicine information were physicians, pharmacy personnel, and package leaflets, while 1 in 8 (n=36, 12%) did not ask for or obtain medicine information during the last year. A total of 9 in 10 participants trusted health professionals as a source of medicine information, while almost half of those using the internet trusted the information they found on the internet regardless of sources. The websites considered most trustworthy were the national health portals and other official health information websites.

To our knowledge, this is the first study investigating internet use to obtain medicine information among Norwegian pharmacy customers. Studies from other countries have reported that the use of the internet for medicine information started increasing during the 2000s [31,33-35] and reached up to 37% in 2019 [6,21]. There seems to be an increasing trend in seeking web-based medicine information, and this is supported by our study. Seeking health information on the internet can improve the patient-physician relationship, involvement in one’s own health, and increase shared decision-making [52].

Age was the only variable associated with internet use to obtain medicine information in this study. Not surprisingly, there is a positive association between being young and using the internet for medicine information. This finding is in line with previous research [9,21,31]. Older persons often consider their physician as their main information source, and they are less aware of other sources [31]. Younger persons may consider it easier to find information by using the internet instead of asking health professionals [31,53]. The Eurobarometer from 2014 identified people aged 40-54 years to be the most frequent users of the internet as a source of health information, with 62% using it daily or almost daily [54]. Medicine information-seeking behavior and the use of medicine information sources are influenced by gender and education [31]. Some patients and specific medicine users use the internet more than the general population. For example, studies have reported that 60% of pregnant women and 68% of patients with chronic conditions use the internet for medicine information [24,43]. We found that regular medicine use could potentially influence internet use for medicine information, but this association was not statistically significant. This could be due to selection bias or a limited number of respondents. The frequency of using the internet for medicine information according to regular medicine use, as well as different medicine user groups, should be analyzed in a larger population sample.

This study demonstrates that almost half of those using the internet for medicine information trust the information they find on the internet. When asked which specific websites they trust, public health portals and other validated sites run by government organizations are considered most trustworthy, but pharmacy sites are also considered trustworthy. A very low proportion trusted general health and lifestyle sites and social media. These results suggest that people are aware of the potential for misinformation on websites, which is consistent with previous research [55]. The Eurobarometer study found that >90% of people using the internet state that they know how to navigate the internet to find information about health-related questions, but 40% did not believe the information source was trustworthy [1]. Recognizing trustworthy sites of information is crucial for an evidence-based approach to health care [56]. One recent systematic review found that the mean quality score across web-based health information sites remained good, but few were very good, and none were excellent [39]. The authors found that information from government organizations ranked highest and was most reliable. As our respondents use and trust national health portals and other official health information sites, they seem to be trusting high-quality websites.

We further found that traditional sources, such as physicians, pharmacy personnel, and packet leaflets, are also used for obtaining medicine information. The physician was the main source. This is expected as the physicians prescribe the medicine and explain why the patient should take the medicine [57]. Pharmacists were the second highest source, which may reflect a need to know how to use the medicine correctly. These findings are in line with other studies reporting medicine information sources [21,30,31,55,57,58]. Our study also found that the proportion obtaining medicine information from pharmacy personnel and package leaflets was higher among younger persons and women than among older persons and men, respectively. Regular medicine users obtained medicine information from health professionals and the package leaflet to a greater extent than respondents with no regular medicines. This is not surprising, as those who use medicine on a regular basis most likely see a physician and collect the medicine at the pharmacy more often. Possibly, they may also need more medicine information. Most of our respondents used 1-3 sources for medicine information, which is in line with previous studies [21,43].

Our respondents reported that they mainly used the internet to obtain information about side effects, how the medicine works, and how it should be used. Previous studies have found that patients are mainly interested in more information about side effects and interactions [57,59,60]. Very few of our respondents sought information about interactions. One reason for this may be that they trust the prescribing physician’s knowledge and that they will receive information if they need to avoid certain medications or foods. Another reason may be that they obtain this information from the pharmacists or pharmacy staff. The leaflets provided with the medication list many possible side effects, while the pharmacists may focus on the main ones to watch out for. Most respondents (n=272, 91%) trusted the information they received from health professionals. This is reassuring since physicians and pharmacy personnel are key counselors on medicine use [43]. As health professionals still are the most preferred and trusted source of information [5,6,55], physicians and pharmacists should continue to provide high-quality medicine information to patients and other medicine users in clinical consultations and encounters at the pharmacy. Health personnel may also help medicine users by providing information that they are unaware they needed.

A higher proportion of health professionals obtained medicine information from physicians, pharmacists, and the internet compared to people who are not health professionals. This was expected as they often handle medicines for patients or clients. Hermes-DeSantis et al [61] have shown that almost 90% of health professionals search the internet for health information daily or several times a week, and the most common questions were about medicines, dosing and administration, drug-drug interaction and adverse events, and safety.

One in 8 of the respondents did not obtain medicine information from any source. A study from Finland has analyzed long-term trends of medicine information sources and found that the proportion of medicine users reporting using no medicine information sources increased more than 7-fold from 4% (77/1944) in 1999 to 28% (467/1671) in 2014. They also demonstrated that medicine information received from physicians declined over time [21]. This trend is worrying as more and more health care services are based on patient involvement and shared decision-making, which requires well-informed patients. More repeated-measure studies should be conducted to monitor this trend. Another worrying trend is the increase in the use of web-based pharmacies where the medicines are delivered to patients by post or courier services. This removes the personal contact between the customers and pharmacy staff, and may reduce the general medicine knowledge among medicine users. Increasing the knowledge about the medicine that one is taking should ensure a better understanding of the purpose and use, and as a result improve adherence, health, and patient satisfaction [45].

### Strengths and Limitations

This study recruited respondents from all but one pharmacy in a medium size municipality in Norway. Demographic variables such as age, gender, and level of education correspond with previous cross-sectional studies involving pharmacy customers [6,55,62,63]. The survey instrument was robust as it was based on questions used in a national repeated survey from Finland [21] implying a high content validity. The questionnaire was piloted to ensure clarity, ease of completion, functionality, and that the length was acceptable. Another strength of this study is that we managed to include the oldest age group. A total of 76 (25%) respondents were older than 70 years, and 30 (10%) were older than 80 years.

The survey was distributed in community pharmacies, and respondents were approached while they were waiting for a prescription or to be served at the counter. The fact that they had the opportunity to complete the questionnaire at the pharmacy might have improved the response rate. However, we were unable to ask all customers, and this might have introduced selection bias. It is possible that persons showing interest were asked more frequently than those who stated that they were busy. Our respondents might also reflect a population more interested in medicine information and the internet in general. All data were self-reported, and the results must be interpreted accordingly.

### Practical Implication

Medicine users trust the medicine information they receive from health professionals and the websites run by health authorities. A low proportion trust the information they find on social media and general health and lifestyle websites. This result is reassuring as it suggests that people are aware of the potential for web-based misinformation.

### Conclusion

The internet is frequently used as a source of medicine information among Norwegian pharmacy customers, but most still obtain information from health professionals and packet leaflets. Medicine users are aware of the potential for web-based misinformation, and they trust websites run by the health authorities.

## Data Availability

The data set used in this study is available from the first author on request.
